# Supplementary material for: Flower and pod development, grain-setting characteristics and grain yield in Chinese milk vetch (Astragalus sinicus L.) in response to pre-anthesis foliar application of paclobutrazol
Source: PLoS One. 2021 Feb 8;16(2):e0245554. doi: 10.1371/journal.pone.0245554 (PMC7870059; doi:10.1371/journal.pone.0245554)
Supplement: Attachment — Submitted filename: Comments.pdf [file pone.0245554.s004.pdf]

Title of the manuscript	: Flower and pod development, grain-setting characteristics and grain yield in Chinese milk vetch (*Astragalus sinicus* L.) in response to pre-anthesis foliar application of paclobutrazol
Manuscript #	: PONE-D-20-26606
Last date for submission	: 20-10-2020

Technical comments:

1. Methods of application, selection of statistical design and growth parameters studies are appropriate.
2. Mention the purity of Paclobutrazol, company name and whether it is commercial or plant cell culture tested reagent? Also mention which surfactant at what concentration was used for foliar application?
3. Since the plant is mainly used as green manure, study or data related to residual analysis of Paclobutrazol should be included. There are reports of negative effects of Paclobutrazol on soil microflora and next crop to be cultivated. (https://www.sciencedirect.com/science/article/pii/S0929139302001105?via%3Dihub; https://link.springer.com/article/10.1007/s11356-019-05947-9)

General comments:

1. Please mention one format of unit i.e. /L or l^{-1} for all the unit types.
2. Sub-heading 3.3 should be corrected as other sub-headings.

Grammatical and typographical errors:

Sr. No.	Line number	Phrase/Word	Correction
1	129	…which away from…	…which was away from…
2	146	…with extracting solution of…	…with an extracting…
3	185	significant	significance
4	191	plant	plants
5	193	plant	plants
6	194	effect	effects
7	327	flower	flowers
8	337	develop	developing

.
