# Supplementary material for: Flower and pod development, grain-setting characteristics and grain yield in Chinese milk vetch (Astragalus sinicus L.) in response to pre-anthesis foliar application of paclobutrazol
Source: PLoS One. 2021 Feb 8;16(2):e0245554. doi: 10.1371/journal.pone.0245554 (PMC7870059; doi:10.1371/journal.pone.0245554)
Supplement: Attachment — Submitted filename: Response to Reviewers.doc [file pone.0245554.s005.doc]

**Dear Editors and Reviewers:**

Thank for your valuable comments on manuscript (PONE-D-20-26606). We have carefully considered the comments and revised the manuscript in detail. The responses to your comments are appended below:

**Comment 1:** Mention the purity of paclobutrazol, company name and whether it is commercial or plant cell culture tested reagent? Also mention which surfactant at what concentration was used for foliar application?

**Response:** We thank for your careful check and have accepted this suggestion. In the revised manuscript, the purity of paclobutrazol, company name and it is commercial has been described and added in detail in L111–113 page 6. The recommended concentration dosage of paclobutrazol was used for foliar spraying on crops was 50~1000 mg· kg^-1^, and on fruit trees was 1000~1500 mg· kg^-1^.

**Comment 2:** Since the plant is mainly used as green manure, study or data related to residual analysis of Paclobutrazol should be included. There are reports of negative effects of paclobutrazol on soil microflora and next crop to be cultivated.

**Response:** We thank for your careful check. The residue of paclobutrazol with different dosage applied to different crops and soil is different. Our research group used gas chromatography-nitrogen and phosphorus detection method to detect the paclobutrazol residue in soil, plant, pod and seed of Chinese milk vetch. It was discovered that at harvest, there was no residue in soil, plant, pod and seed of Chinese milk vetch after using applying recommended dosage（200~600 mg·L^-1^）of 15% paclobutrazol WP once at squaring stage (prior to anthesis). Because Chinese milk vetch is one green manure, so we speculate the reason may be the Chinese milk vetch and ploughing down milk vetch could promote the degradation of paclobutrazol residue by soil microorganisms, the specific reasons need to be further explored.

**Comment 3:** Please mention one format of unit i.e. /L or l^-1^ for all the unit types.

**Response:** We thank for your careful check and have accepted this suggestion. The format of unit "/L " has been replaced by " l^-1^" for all the unit types in the revised manuscript.

**Comment 4:** Sub-heading 3.3 should be corrected as other sub-headings.

**Response:** We thank for your careful check. The sub-heading 3.3 has been corrected as other sub-headings in the revised manuscript.

**Comment 5:** Line 129: The authors indicate that "….which away from…." the phrase should be: "….which was away from….".

**Response:** We thank for your careful check and have accepted this suggestion. This phrase has been changed to “….which was away from….” in line 131 page 6 in the revised manuscript.

**Comment 6:** Line 146: The authors indicate that "….with extracting solution of…." the phrase should be: "….with an extracting solution of….".

**Response:** We thank for your careful check. This phrase has been changed to “….with an extracting solution of….” in line 148 page 7 in the revised manuscript.

**Comment 7:** Line 185: The authors indicate that "….significant…." the word should be: "….significance….".

**Response:** We thank for your careful check. The word has been revised to “…. significance….” in line 187 page 9 in the revised manuscript.

**Comment 8:** Line 191: The authors indicate that "….plant…." the word should be: "….plants….".

**Response:** We thank for your careful check. The word has been revised to “…. plants….” in line 193 page 9 in the revised manuscript.

**Comment 9:** Line 193: The authors indicate that "….plant…." the word should be: "….plants….".

**Response:** We thank for your careful check. The word has been revised to “…. plants….” in line 195 page 9 in the revised manuscript.

**Comment 10:** Line 194: The authors indicate that "….effect…." the word should be: "….effects….".

**Response:** We thank for your careful check. The word has been revised to “…. effects….” in line 196 page 9 in the revised manuscript.

**Comment 11:** Line 327: The authors indicate that "….flower…." the word should be: "….flowers….".

**Response:** We thank for your careful check. The word has been changed to “…. flowers….” in line 330 page 16 and modified in all text in the revised manuscript.

**Comment 12:** Line 337: The authors indicate that "….develop…." the word should be: "….developing….".

**Response:** We thank for your careful check. The word has been changed to “…. developing….” in line 340 page 16 and modified in all text in the revised manuscript.

Other changes:

1. In the revised manuscript, we added an author “Wei Ren”who was contributed a lot to the analysis of experimental data, and added this author’s list and affiliations and contribution.

Yours sincerely,

Chunzeng Liu
